# P-1990. RNA Sequencing Identifies Antimicrobial Resistance Genes Directly from Peripheral Blood of Patients

**DOI:** 10.1093/ofid/ofaf695.2157

**Published:** 2026-01-11

**Authors:** Gerard J Nau, Jaewook Shin, Brandon E Armstead, Alfred Ayala, Maya Cohen, William Fairbrother, Alger M Fredericks, Mitchell M Levy, Emanuele Raggi, Kwesi Lillard, Gregory Jay, Sean F Monaghan

**Affiliations:** Rhode Island Hospital/Brown University Health, Providence, Rhode Island; Rhode Island Hospital/Brown University Health, Providence, Rhode Island; Rhode Island Hospital/Brown University Health, Providence, Rhode Island; Rhode Island Hospital/Brown University Health, Providence, Rhode Island; Rhode Island Hospital/Brown University Health, Providence, Rhode Island; Brown University, Providence, Rhode Island; Rhode Island Hospital/Brown University Health, Providence, Rhode Island; Rhode Island Hospital/Brown University Health, Providence, Rhode Island; Rhode Island Hospital/Brown University Health, Providence, Rhode Island; Rhode Island Hospital/Brown University Health, Providence, Rhode Island; Rhode Island Hospital/Brown University Health, Providence, Rhode Island; Rhode Island Hospital/Brown University Health, Providence, Rhode Island

## Abstract

**Background:**

Prompt administration of antimicrobials improves sepsis outcomes. Molecular testing is transforming infectious disease diagnostics, but direct-from-blood identification of pathogens and antimicrobial resistance (AMR) has lagged. In this study, we tested the hypothesis that AMR genes could be identified in the peripheral blood of patients using RNA sequencing (RNA-seq).

**Methods:**

A single center, prospective study was conducted of patients with sepsis in the intensive care unit (ICU) and with suspected infection in the emergency department (ED). Whole blood samples from ICU patients were drawn in PAXgene tubes on hospital day 0, 1, 3, and 7 and from ED patients at presentation. Deep RNA-seq of at least 100 million reads per sample was performed. Reads not mapped to human genome assembly GRCh38 (“unmapped” reads) were aligned to a custom AMR genome created from the Comprehensive Antibiotic Resistance Database (CARD). Command line *bash* and *awk* script and RStudio were utilized for computational processing.

**Results:**

A total of 274 patients, 221 from the ED and 53 from the ICU, were included. This yielded 329 RNA-seq samples including data from multiple days, amounting to 3.3 billion reads. Of the 329 samples, 180 (54.7%) had no reads that aligned to the AMR genome while 149 (45.3%) had at least 2 reads that aligned. The 149 AMR-positive samples had a total of 3648 reads, with a median of 6 reads per sample (IQR: 12). When stratified by resistance mechanisms, most of the reads aligned to AMR genes coding for antibiotic efflux pumps (59.6%), followed by antibiotic inactivation (26.5%), target replacement (11.0%), target protection (2.2%), and reduced antibiotic permeability (0.7%).

**Conclusion:**

Efflux pumps may be an underappreciated mechanism of AMR. Their expression leads to fitness costs, leading to tightly regulated (over)expression. This could cause a discordance between *in vivo* expression and *in vitro* where AMR testing is performed. More generally, RNA sequencing can identify AMR genes in peripheral blood, a dynamic assessment of expression since the half-life of RNA is short. Importantly, RNA will reflect AMR phenotypes better than DNA since mRNA is one step nearer to protein production.

**Disclosures:**

Sean F. Monaghan, MD, Alcini, LLC: Ownership Interest

